# Clinical proteomics for liver disease: a promising approach for discovery of novel biomarkers

**DOI:** 10.1186/1477-5956-8-70

**Published:** 2010-12-31

**Authors:** Hirofumi Uto, Shuji Kanmura, Yoichiro Takami, Hirohito Tsubouchi

**Affiliations:** 1 Department of Digestive and Lifestyle-related Diseases, Health Research Human and Environmental Sciences, Kagoshima University Graduate School of Medical and Dental Sciences, Kagoshima, 890-8544, Japan

## Abstract

Hepatocellular carcinoma (HCC) is the fifth most common cancer and advanced hepatic fibrosis is a major risk factor for HCC. Hepatic fibrosis including liver cirrhosis and HCC are mainly induced by persistent hepatitis B or C virus infection, with approximately 500 million people infected with hepatitis B or C virus worldwide. Furthermore, the number of patients with non-alcoholic fatty liver disease (NAFLD) has recently increased and NAFLD can progress to cirrhosis and HCC. These chronic liver diseases are major causes of morbidity and mortality, and the identification of non-invasive biomarkers is important for early diagnosis. Recent advancements in quantitative and large-scale proteomic methods could be used to optimize the clinical application of biomarkers. Early diagnosis of HCC and assessment of the stage of hepatic fibrosis or NAFLD can also contribute to more effective therapeutic interventions and an improve prognosis. Furthermore, advancements of proteomic techniques contribute not only to the discovery of clinically useful biomarkers, but also in clarifying the molecular mechanisms of disease pathogenesis by using body fluids, such as serum, and tissue samples and cultured cells. In this review, we report recent advances in quantitative proteomics and several findings focused on liver diseases, including HCC, NAFLD, hepatic fibrosis and hepatitis B or C virus infections.

## Introduction

Diagnostic methods for hepatocellular carcinoma (HCC) include imaging, such as abdominal ultrasonography and computed tomography (CT), and measurement of serum tumor markers. Alpha-fetoprotein (AFP), AFP lectin fraction L3 (AFP-L3), and des-gamma-carboxy prothrombin (DCP, also known as PIVKA-II) are widely used clinically as serum tumor markers of HCC. However, the sensitivity of AFP or DCP for detecting early stage HCC is only 30-60% [1-4]. Although combination measurements of AFP and DCP can improve the diagnostic performance, the diagnostic accuracy is still low for HCC lesions of ≤2 cm. Therefore, the development of a new diagnostic method for early stage HCC is needed to improve outcomes [5-7].

The main cause of liver cirrhosis and HCC is persistent hepatitis B or C virus infection. The degree of hepatic fibrosis is associated with the occurrence of HCC, and serum hyaluronic acid and type IV collagen levels are used for diagnosis of hepatic fibrosis including cirrhosis, but these markers do not always reflect the stage of hepatic fibrosis assessed by liver biopsy [8,9]. In addition, the incidence of nonalcoholic fatty liver diseases (NAFLD) has increased worldwide, but no specific biomarker is available and invasive liver biopsy is still required for definite diagnosis of NAFLD, especially for nonalcoholic steatohepatitis (NASH), which can progress to cirrhosis and HCC [10,11]. Therefore, there is a need to identify blood (serum or plasma) markers that are specific for early diagnosis of HCC, prediction of carcinogenesis from liver cirrhosis, progression of liver cirrhosis, and diagnosis of NASH. These analyses may also aid in the elucidation of the mechanism(s) underlying the pathogenesis of hepatitis and hepatocarcinogenesis.

Proteomics is the term used for exhaustive analysis of protein structure and function in an organ or tissue. The levels of gene expression and protein production are not necessarily proportional, and protein activity is frequently regulated by posttranslational modifications such as phosphorylation [12,13]. Proteomics is useful for elucidation of the pathology and discovery of disease markers for HCC and chronic liver diseases. Serum and plasma are readily used as clinical samples since they can be obtained using less invasive methods. If a biomarker associated with the pathology, disease progression or efficacy of treatment is identified in serum or plasma, it can be easily applied for early or differential diagnosis of diseases. Recent advances in methods for mass spectrometric analysis, including protein labeling and amino acid analysis, facilitate highly sensitive and exhaustive proteomic analysis of patient samples. These advances in proteomics techniques have promoted exploration of biomarkers for malignant tumors including HCC and for chronic liver diseases including liver cirrhosis, NAFLD and chronic hepatitis B or C. In this review, we provide an overview of recent findings in proteomic analysis of those liver diseases.

## A - Clinical proteomics

For the efficient discovery of biomarkers, more quantitative and reproducible techniques are required. Therefore, differential analysis of protein expression is frequently used in clinical proteomics. Quantitative proteomic approaches can be separated into both labeling (Table 1) and labeling-free methods (Table 2), and the labeling method is separated into gel-based and non-gel-based methods. The most typical method of the gel-based differential approach is two-dimensional fluorescence difference gel electrophoresis (2D-DIGE) [14,15]. On the other hand, non-gel-based methods include some stable isotope-labeling methods, such as cleavable isotope-coded affinity tags (cICAT) [16], stable isotope labeling by amino acids in cell culture (SILAC) [17,18], 2-nitrobenzenesulfenyl (NBS) labeling [19] and protein quantitation using isobaric tags for relative and absolute quantitation (iTRAQ) [20]. In addition, labeling-free methods; surface enhanced laser desorption ionidization (SELDI) methods [21] and ClinProt^® ^systems [22] based on affinity-column or -beads chromatographic methods were beneficial to analyze the blood samples (Table 2). Moreover, molecular information can be obtained from comparison of multiple samples in a single analysis with these methods. The techniques of separation and detection on mass spectrometric analysis and molecular identification have also progressed with improvement in accuracy. The development of high-sensitivity, high-throughput, and exhaustive analytical methods has facilitated identification of trace proteins in biological samples, and clinical proteomics is now performed using new protein analysis techniques. However, these are mostly basic studies, rather than disease-based proteomics useful for bedside diagnosis and prediction of therapeutic effects. Thus, proteomics studies of the association between clinical data and results obtained from cells, tissues and clinical samples are required.

**Table 1:** Quantitative proteomic techniques that have been applied to clinical proteomics using labeling method

Methods	Type of method	Labeling reagents	Interests	Comparable number of samples/assay	References
2D-DIGE	Gel-based	Cy2, Cy3, Cy5, IC3-OSu, IC5-OSu	Most frequently used gel-based method	2 samples	[14], [15]

cICAT	Non-gel based	^12^C-ICAT (light) ^13^C-ICAT (heavy) Labeled to cysteine thiol group	Most frequently used isotope labeling method	2 samples	[16]

SILAC	Non-gel based	^12^C- or ^14^N-lysine and arginine (light) ^13^C- or ^15^N-lysine and arginine (heavy) Incorporated into cultured cells	Pre-labeling method. Cell lysates and conditioned media can be analyzed.	2 samples	[17], [18]

NBS	Non-gel based	^12^C-NBS (light) ^13^C-NBS (heavy) Labeled to tryptophan indole group	Simple MS spectra can be obtained because there is less tryptophan in protein sequences.	2 samples	[19]

iTRAQ	Non-gel based	Isobaric tags (m/z 305, in total) (m/z, reporter) + (m/z, balancer): (113) + (192), (114) + (191), (115) + (190), (116) + (189), (117) + (188), (118) + (187), (119) + (186), (121) + (183) Labeled to lysine amino group	Expression ratio can be used to quantify the signal intensity of reporter peaks. Many samples can be assayed in one experiment.	2 ~ 8 samples	[20]

2D-DIGE; two-dimensional fluorescence difference gel electrophoresis, cICAT; cleavable isotope-coded affinity tags, SILAC; stable isotope labeling by amino acids in cell culture, NBS; 2-nitrobenzenesulfenyl, iTRAQ; isobaric tags for relative and absolute quantitation, Cy; cyanine,

**Table 2:** Quantitative proteomic techniques that have been applied to clinical proteomics using labeling-free method

Methods	Types of chips or magnetic beads	Interests	References
ProteinChip SELDI	IMAC30 (metal modified), CM10 (cation exchanging), WCX2 (weak cation exchanging) Q10 (anion exchanging), H50 (reverse-phase), H4 (reverse-phase), NP20 (normal-phase), Gold	Analyses: a few μl of serum/plasma (without removal of abundant proteins), urine, cell/tissue lysates and conditioned media Identification: MS/MS, LC-MS/MS	[21]
		
ClinProt^®^	Profiling: WCX, WAX, HIC8, IMAC-Cu Large-protein beads: HIC1, HIC3 Peptides beads: HIC18 Phospho beads: IMAC-Fe Glyco beads: LAC ConA, ConAC boronic Antibody capture beads: IAC ProtG	Performance: Many samples can be assayed in parallel.	[22]

SELDI; surface enhanced laser desorption ionidization, MS; mass spectrometry, LC; liquid chromatography

## B - Proteomic analysis of hepatocellular carcinoma

### 1 - Serum proteomics in patients with HCC

Protein separation by 2-dimensional electrophoresis (2-DE) is a well-established and widely used method with easy handling and good reproducibility. In a 2-DE study of protein expression in sera of 5 patients with HCC (2 hepatitis B virus surface [HBs] antigen-positive cases, 2 hepatitis C virus [HCV] antibody-positive cases, and one case negative for both] and healthy subjects, 317 proteins were separated and identified, of which 6 (annexin VI isoform 1, complement component 9, ceruloplasmin, and serum amyloid A4, A2 and A1 isoform 2) were proposed as diagnostic markers for HCC [23]. In sera of patients with hepatitis B virus (HBV)-related HCC investigated by 2-DE, 8 proteins with significant differences in expression levels compared to controls were identified (transferrin, transthyretin, α1-antitrypsin, clusterin, haptoglobin α2 chain, ceruloplasmin, heat-shock protein 27 [HSP27], and α-fetoprotein), and HSP27 was positive in 90% of the HCC cases, showing its value for HCC screening [24]. Useful diagnostic markers may be discovered in proteins directly identified by 2-DE separation of patient serum, followed by extraction of protein spots from the gel and identification by peptide-mass fingerprinting (PMF) and MS/MS analysis. However, albumin, globulin, transferrin, and antitrypsin account for about 90% of the serum protein composition, and the large amounts of these proteins interfere with separation of serum proteins by 2-DE, and make separation and analysis difficult. To analyze serum using 2-DE, removal of these abundant proteins and subsequent detection of changes in trace protein levels are necessary. Ang et al. removed albumin by pretreatment of sera from patients with HCC and chronic liver disease (CLD) using lectin and then compared the glycosylated haptoglobin expression level using 2-DE [25]. Expression of glycosylated haptoglobin was increased in the HCC group, and the level was higher in advanced HCC compared to early stage HCC, suggesting that glycosylated haptoglobin is useful for diagnosis and prediction of the HCC stage [25]. These findings also suggest that pretreated serum is better than non-treated serum for 2-DE analysis.

There are two methods of serological diagnosis using the ProteinChip SELDI system: one uses identification of individual proteins and functional analysis, and the other is based on a classification (decision tree) method established by data mining without protein identification. Identification of a protein corresponding to a target peak is difficult using the ProteinChip SELDI system, and the classification-based diagnostic method (multi-marker analysis) is frequently used, in which identification of each protein corresponding to an individual peak is not necessary. The disease and control groups are differentiated based only on the expression levels of several protein peaks. We established a classification method based on 7 peaks that were highly distinguishable between HCV-related HCC and HCV-related CLD, and showed that this method is applicable for diagnosis of both early stage and advanced HCC [26]. This approach was capable of detecting HCC earlier than detection of tumorous lesions by abdominal ultrasonography, and was more useful for early diagnosis than current tumor markers such as AFP and DCP. Similarly, Zinkin et al. developed a diagnostic method using 11 protein peaks detected by the ProteinChip SELDI system, and found a sensitivity and specificity for diagnosis of HCV-related HCC of 79% and 86%, respectively [27]. The diagnostic sensitivity and specificity do not differ significantly from those of methods using current HCC markers (AFP, AFP-L3 fraction, and DCP), but the performance for diagnosis of small HCC of ≤2 cm was better than that for methods using current markers.

He et al. selected 3 protein peaks (5890, 11615, and 11724 Da) in serum that showed significant differences in HBV-related HCC patients compared to HBV patients without HCC, and found that HBV-related HCC could be diagnosed in almost 100% of cases based on these proteins [28]. The SELDI method was combined with 2-DE to identify the protein with a peak at 11615 Da as serum amyloid A (SAA). However, the positive rate was also high in patients with HBV-related CLD in this analysis, indicating that the method is not specific for HCC. Cui et al. reported that patients with HBV-related HCC or CLD could be distinguished from healthy subjects by multi-marker serum analysis with sensitivity and specificity of 90% or higher [29], but it is doubtful whether this method could be used for differentiation of HCC from CLD. Similarly, sensitivity of 100% and specificity of 92 or 97% have been reported for diagnosis of HBV-related HCC [30], but the control group consisted of healthy subjects without CLD and the utility for early diagnosis of HCC in patients with cirrhosis is doubtful. Göbel et al. established a method for differentiation of HCV-related HCC from liver cirrhosis without HCC using 4 protein peaks at 7486, 12843, 44293, and 53598 Da (multi-marker analysis), and found that the method was useful for diagnosis of early stage HCC [31]. Ward et al. also reported a multi-marker analysis with 94% sensitivity, 86% specificity, and 0.92 AUROC [32]. Thus, multi-marker analysis is useful for diagnosis. However, a ProteinChip SELDI system is necessary for analysis, and this system is expensive compared with commercially available kits such as those for ELISA, and not all facilities can use this system. Moreover, the peak protein intensity detected by a protein chip system may vary among analytical devices and facilities, and further technical advances are needed for clinical application of multi-marker analysis for diagnosis of early stage HCC (Table 3).

**Table 3:** The peaks detected by ProteinChip SELDI in patients with hepatocellular carcinoma

Subjects	Protein/Peptide Peaks (m/z)	Type of Protein Chip	Identification	References
HCV-related HCC *vs*. non-HCC	3444, 3890, 4067, 4435, 4470, 7770	CM10	ND	[26]

HCC *vs*. non-HCC (cirrhosis)	3687, 3906, 26457 11853, 11873, 11887, 13391 11319, 17783, 17906, 18021	CM10, IMAC30, H50,	13391-Da; Cystatin C	[27]

HBV-related HCC *vs*. non-HCC	5890, 11615, 11724	IMAC30-Cu	ND	[28]

CHB LC HCC (*vs*. Healthy control)	22842 (up), 2957, 2049 (down), 2049 (up), 3166 (down), 23381, 28040 (up), 2018 (down)	WCX2	ND	[29]

HBV-related HCC *vs*. Healthy control	7777, 9250, 16200	WCX2	ND	[30]

HCV-related HCC *vs*. HCV-related LC	2873, 6646, 7775, 10525, 67867	CM10	6646-Da; Apolipoprotein C-I	[31]

HCV-related HCC *vs*. HCV-related LC	22960, 23530	IMAC30	ND	[32]

SELDI; surface-enhanced laser desorption and ionization mass spectrometry, HCV; hepatitis C virus, HCC; hepatocellular carcinoma, HBV; hepatitis B virus, CHB; chronic hepatitis B, LC; liver cirrhosis, ND; not done,

In analysis using cICAT and liquid chromatography-electrospray ionization tandem mass spectrometry (LC-ESI-MS/MS), Kang et al. compared serum proteins between 9 cases of HCC and 9 cases of liver cirrhosis, and identified 31 proteins with differences in expression levels. Of these proteins, significantly enhanced expression of α1-acid glycoprotein (AGP) was observed in the HCC validation group (HCC; N = 52, liver cirrhosis; N = 40), showing that AGP is a candidate serum diagnostic marker for HCC [33]. Thus, serum proteomic analysis using a combination of cICAT and LC-ESI-MS/MS can be used for direct identification of potential protein markers.

AFP and DCP are frequently used in current diagnosis of HCC. These proteins are directly expressed by tumors and their blood levels are reduced by local treatment or tumor resection. Therefore, they serve as indices for diagnosis, therapeutic effect and recurrence. In contrast, the proteins identified by serum protein expression analysis described above are not necessarily produced by the tumor (for example, they may be produced by immune cells that act on tumor cells) and this may be limitation of current biomarker exploration by serum analysis. Moreover, some proteins in HCC change with progression of the pathology of the underlying diseases of chronic hepatitis and liver cirrhosis. Therefore, specificity is of importance in clinical proteomic analysis using serum from patients with HCC.

### 2 - Tissue proteomics in patients with HCC

HCC tissue has been widely used in proteomics because a large amount of tissue can be obtained relatively easily from surgical specimens [34-41]. In a study in which protein expression in liver tissue was investigated by 2-DE in 40 cases of HBV-related HCC and 36 control subjects (20 patients with liver cirrhosis and 16 normal liver tissues from residual grafts of liver donors), 14 proteins with ≥2-fold changes in expression level were identified in patients with HCC compared to the controls [34]. Of these proteins, vimentin expression was significantly elevated in the HCC group. The vimentin level was also elevated in serum from the HCC patients, which was useful for diagnosis of HCC lesions of ≤2 cm. Comparison of HCC and normal liver tissues using 2D-DIGE has shown reduced expression levels of proteins associated with metabolism and increased expression of stress-related proteins of the HSP family in HCC, with aldo-keto reductase 1C2, thioredoxin, and transketolase proposed as HCC markers [35,36]. Luk et al. analyzed liver tissue of 146 patients by 2-DE+MS/MS and detected 1800 protein spots. Three of these protein spots with strong expression in HCC were identified as Hsp27, Hsp70 and glucose-regulated protein (GRP) 78. Hsp27 was found to be highly correlated with AFP, and GRP78 was associated with venous tumor invasion [37]. Sun et al. performed protein expression analysis to search for HCC biomarkers using liver tissue samples from 59 patients with liver diseases (39 with HCC and 20 with liver cirrhosis) and 16 healthy subjects, and 63 plasma samples (35 patients with HCC, 16 with liver cirrhosis, and 12 healthy subjects). In both liver tissue and plasma, lamin B1 (LMNB1) expression was significantly enhanced in HCC patients compared to healthy subjects, and the LMNB1 expression level was associated with the cancer stage, leading to the conclusion that this protein is a useful biomarker for early stage HCC [38]. An increased clathrin heavy chain level and a lower formiminotransferase cyclodeaminase level in 2-DE analysis of HCC tissue have also been proposed to be useful for diagnosis of early stage HCC [39], and APC-binding protein EB1 expression in resected HCC specimens has been related to the survival and recurrence rates after resection [40].

Use of whole resected liver specimens containing HCC for proteomic analysis allows detection of proteins expressed not only by cancer cells, but also by cells infiltrating around the tumor and by interstitial cells. Selection of cancer tissue by laser capture microdissection (LCM) followed by 2-dimensional liquid chromatography tandem mass spectrometry (2D-LC/MS-MS) can be used to identify proteins that differ quantitatively between disease and control tissue [41]. This approach is useful for analysis of surgical samples and may help to improve the understanding of the mechanism of carcinogenesis in HCC.

## C - Proteomic analysis of nonalcoholic fatty liver disease (NAFLD), hepatic fibrosis and liver cirrhosis

### 1 - Serum proteomics of NAFLD, hepatic fibrosis and liver cirrhosis

The number of cases of NAFLD including NASH has shown a recent increase, and NASH is a risk factor for HCC. NASH has a pathology similar to that of alcoholic liver diseases and is accompanied by inflammation and fibrosis that progresses to liver cirrhosis and HCC. Excess nutrition, obesity, insulin resistance, and oxidative stress are thought to be involved in the development and progression of NASH, but the molecular mechanisms remain uncertain. About 30% of subjects in a health check-up in Japan showed abnormalities in serum markers of liver function such as ALT, AST and γ-GTP, and most cases were assumed to be NAFLD. Identification of patients with NASH among those with NAFLD is very important for prevention of liver cirrhosis and HCC through intensive treatment. However, invasive liver biopsy is currently required for diagnosis of NASH, since no specific serum marker is available for use in a noninvasive test [10]. In serum proteomics in patients with NAFLD, four apolipoproteins and CD5 antigen-like protein (CD5L) were identified by 2-DE analysis. Of these proteins, CD5L reflects the severity of hepatic fibrosis in NAFLD and its serum level increases in cases with severe fibrosis, suggesting that it may serve as a diagnostic marker of NASH [42]. An analysis of sera of patients with NAFLD using the ProteinChip SELDI system identified 4 protein peaks with significant changes in patients with NASH compared to obese patients without NAFLD [11]. These peaks may be useful for diagnosis of NASH, but the corresponding proteins have yet to be identified. Interestingly, mRNA expression in liver tissue was analyzed using a microarray in the same patients in this study, and this analysis may advance the understanding of the molecular mechanism of development and progression of NAFLD. Currently, the association between serum protein expression and gene expression in liver tissue is unclear.

Bell et al. analyzed sera of 85 patients with NAFLD, including cases of NASH and simple fatty liver by LC/MS-MS and identified 1738 proteins, of which 9 reflected differences in fibrosis among the NASH patients and 21 were proposed as useful biomarkers to distinguish NASH (F3/F4) with advanced fibrosis from simple fatty liver [43]. Unfortunately, no single protein for discrimination between simple fatty liver and NASH was obtained in this analysis. A panel diagnostic method using fibrinogen β chain, retinol binding protein 4, serum amyloid P component, lumican, transgelin 2, CD5L, complement component C7, insulin-like growth factor acid labile subunit, and transgelin 2 has been developed that discriminates among healthy subjects, patients with simple fatty liver, and patients with NASH with high power. In this report, serum was separated by nano-HPLC in proteomic analysis, and proteins were identified and quantified by electrospray ionization (ESI) [43]. Such combination method has high-resolution and relatively favorable quantitative performance with a very small amount of sample, therefore is capable of identifying many proteins, which has led to expansion of its use. However, whether the identified proteins are truly specific to NAFLD and useful for diagnosis of NASH remains to be investigated.

Proteomic analysis in patients with liver cirrhosis has also been performed to search for hepatic fibrosis markers. Poon et al. developed a scoring system for diagnosis of hepatic fibrosis using 10 factors: 7 protein peaks detected using the ProteinChip SELDI system, and the ALT, total protein, and bilirubin levels in serum. Using this approach, liver cirrhosis was diagnosed with 94% sensitivity and 91% specificity [44]. Morra et al. also showed that a differentiation method based on 8 protein peaks was more useful for evaluation of hepatic fibrosis than the existing Fibro Test (an method that uses α2-macroglobulin, haptoglobin, apolipoprotein A1, total bilirubin, and γ-GTP) [45]. Göbel et al. used 5 serum protein peaks in development of a multi-marker method to differentiate between cirrhotic and non-cirrhotic livers (F1 and F2), and showed that this method could be used to diagnose liver cirrhosis with 80% sensitivity and 67% specificity [31].

In 2-DE analysis of sera from patients with HCV-related CLD, the levels of inter-alpha-trypsin inhibitor heavy chain H4 (ITIH4) fragments, α1-antichymotrypsin, apolipoprotein L1, prealbumin, albumin, paraoxonase/arylesterase 1, and zinc-α2-glycoprotein were reduced and those of CD5 antigen-like protein (CD5L) and β2 glycoprotein I (β2GPI) were elevated in the liver cirrhosis group [9]. Using a similar method, the levels of Mac-2-binding protein, α2-macroglobulin and hemopexin were found to be elevated and those of α1-antitrypsin, leucine-rich α2-glycoprotein and fetuin-A were decreased in advanced liver fibrosis [46]. Identification of serum protein peaks that are altered in liver cirrhosis and verification of their utility in the diagnosis of liver cirrhosis may lead to the discovery of novel diagnostic markers. Callewaert et al. have also recently reported an interesting glycoproteomics approach in developing the methodology for a DNA sequencer-based total serum protein-linked N-glycans [47]. Their methodology allows for high-throughput fingerprinting and sequencing of N-glycans that are present on picomolar amounts of glycoproteins. Using this method, they compared the serum protein-linked N-glycan profiles from compensated cirrhotic and non-cirrhotic chronic liver disease patients, to successfully distinguish the pathogenesis of both disease populations with 79% sensitivity and 86% specificity [48].

### 2 - Tissue proteomics of NAFLD, hepatic fibrosis and liver cirrhosis

To predict the progression of NASH and/or hepatic fibrosis, it is important to gain a better understanding of the pathogenesis and molecular mechanism(s) responsible. To understand the pathogenesis of NAFLD, tissue proteomics is also considered to be a more effective tool. Younossi et al. constructed a model to predict NASH and advanced hepatic fibrosis based upon protein microarray-phosphoproteomics using liver biopsy samples, blood samples and visceral adipose tissue [49]. In this model, using the parameters of age, race, gender, diabetes status, AST, phosphorylated-Akt (Ser 473) and phosphorylated-insulin receptor substrate 1 (IRS1) (Ser 612), it was possible to predict NASH with AUC = 0.860 (81.3% sensitivity and 87.0% specificity) [49]. Charlton et al. compared the protein expression profiles in four groups of liver tissue samples (obese normal, simple steatosis, NASH-mild [inflammation grade 1, fibrosis stage 0-1] and NASH-progressive group [fibrosis stage 2-4]) from obese patients using the combination of iTRAQ with LC-MS/MS [50]. They identified a total of 1362 hepatic-expressed proteins, and found that a 40-kDa keratin sulfate proteoglycan was significantly overexpressed in a progressive manner in NASH (-mild and -progressive), whereas, fatty acid binding protein-1 (FABP-1) was underexpressed in both states of NASH [50].

Several groups have performed proteomic analyses using liver tissue from patients with HCV-related chronic liver disease. Diamond et al. performed a quantitative proteomic analysis of HCV-infected human liver tissue from patients at different stages of fibrosis using ^16^O/^18^O stable isotope labeling combined with the accurate mass and time tag approach, and revealed that 210 of 1641 proteins, including those associated with carbohydrate and fatty acid metabolism and the mitochondrial oxidative stress response, exhibited statistically significant differences that were associated with the fibrosis stage [51]. Mölleken et al. compared the protein expression in hepatocytes and cells from the cirrhotic septa of patients with end-stage liver disease associated with HCV infection at the time of liver transplantation using 2-DE-LC-MS/MS [52]. Several structural proteins were upregulated in cells from fibrotic septa, which were assumed to have arisen from activated hepatic stellate cells. One of these identified proteins, microfibril-associated protein-4 (MFAP-4) was subsequently measured in serum, and were shown to increase as the fibrosis stage increased. Although this marker was not able to discriminate between mild and moderate fibrosis, MFAP-4 was more useful to diagnose cirrhosis associated with HCV infection compared with alcoholic liver cirrhosis. In addition, this report could extend the concept of tissue proteomics into the discovery of serum biomarkers.

## D - Proteomic analysis of hepatitis B or C virus infection

HBV and HCV can induce both acute and chronic necroinflammatory liver disease, and chronic infection with both viruses has a very high risk of developing into HCC. Thus, biomarkers reflecting the pathogenesis of viral infection and/or chronic hepatitis are also necessary to elucidate new potential therapeutic approaches. However, reports of biomarkers that can predict viral infection or the mechanism of hepatitis have not been fully investigated.

He et al. compared sera from normal, HBV infected low- and high-necroinflammatory scoring patients using 2-DE, and identified that the expression of seven proteins, haptoglobin β and α2 chain, apolipoprotein A-I and A-IV, α1-antitrypsin, transthyretin and DNA topoisomerase IIβ correlated with the HBV necroinflammatory scores [53]. More recently, Ren et al. performed a serum proteomic analysis of HBV infection. They compared the changes in serum proteins in patients with acute-on-chronic liver failure (AoCLF) with those in normal subjects or in patients with chronic hepatitis B using 2-DE, and identified 12 of 23 differentially expressed proteins [54]. In this analysis, serum levels of α1-acid glycoprotein was one of the proteins that were significantly decreased in patients with AoCLF [54]. Chen's group performed several *in vitro *proteomic investigations of HBV-infected HepG2 hepatoma cells to evaluate the protein changes associated with virus infection. Using the combined methods of iTRAQ with 2D-LC-MS/MS, they compared the protein expression in non-infected HepG2 with HBV-infected HepG2 cells to identify several proteins that were down-regulated in HBV-infected cells, including S100A6 and annexin A2 [55,56]. On the other hand, the influence of HCV infection is often assessed *in vitro *using the HCV replicon system [57]. Jacobs et al. performed a large-scale proteome analysis of the Huh-7.5 cell line, containing a full-length HCV replicon with the multidimensional LC-MS/MS technique [58]. Then, they identified 4,200 proteins, including lipid metabolism-related proteins, expressed in Huh-7.5 cells. A total of 1,500 proteins were also detected from liver biopsies from HCV-infected patients. More recently, Singaravelu et al. utilized a unique labeling probe, a non-directed phenyl sulfonate ester probe, PS4, which was labeled to a nucleophilic residue within the active site of the enzyme molecules to profile the alteration of activity levels during HCV replication during Huh-7 HCV subgenomic replicon [59]. Nineteen active proteins including protein disulfide isomerase-associated 4, heat shock 70 kDa protein 5 were then identified by 2-DE-LC-MS/MS. Thus, proteomic analysis using HCV replicon is thought to be useful for understanding the mechanism of HCV infection and replication.

## E - Prospects for proteomics in liver diseases

**Figure 1:**
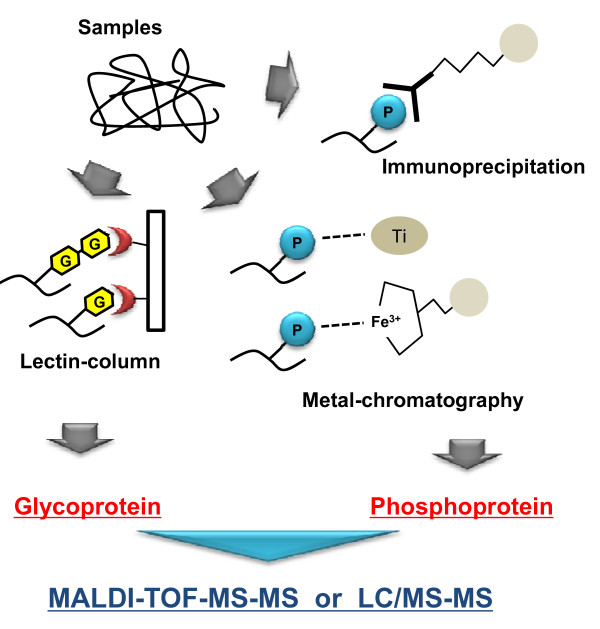
**The outline for identification of glycol- and phosphor-protein**.

Analysis of phosphorylated or glycosylated peptides and proteins is increasingly important in biomarker studies [60]. In addition to identification and localization of modified sites, analysis of their variation may provide important clues to complex biological functions and for exploration of disease biomarkers and new drug development. The outline of proteomic analysis for phosphor- and glyco-ptrotein was shown in Figure 1. Plectin-1 (phospho-Ser-4253) and alpha-HS-glycoprotein (phospho-Ser 138 and 312) have been identified as biomarkers of HCC in an analysis targeting phosphorylated proteins [61].

Differences in carbohydrate chains bound to the same protein in cancer and normal cells are well known, and proteomics focusing on differences in glycosylation of proteins has been performed [62-65]. Different glycosylation patterns of proteins in HCC tissue and plasma have been reported in a study in which HCC samples were pretreated with lectin-bound agarose and the resulting glycoprotein was analyzed by 2D-DIGE and MALDI-TOF/MS. Analysis of plasma by nano-LC/MS-MS showed increased expression of human liver carboxylesterase 1 (hCE1) in HCC patients [66]. Block et al. showed that the hyperfucosylated Golgi Protein 73 (GP73) was elevated in the serum of patients with HCC based upon targeted glycoproteomics using the combined method of HPLC with 2-DE [67]. Lee et al. labeled proteins in the plasma of HCC patients and healthy controls using iTRAQ and identified 14 high-level N-linked sugar chains in the HCC group. Two of the associated proteins were identified as vitronectin (Asn-169, 242) and antithrombin III (Asn-225), and the changes in the sugar chains were proposed as potential markers of HCC [68]. In another study, the level of fucosylated α1 acid glycoprotein (AGP) was found to be higher in patients with HBV-related HCC compared to controls, although there was no significant difference in the total AGP level in serum between the patients and controls [64]. Peptidomics targeting low-molecular-weight peptides [69] and fragmentomics targeting protein fragments [70,71] may also be useful in the search for liver disease markers.

Paradis et al. used the ProteinChip SELDI system to analyze sera from 96 patients with chronic hepatitis C who were treated with interferon and ribavirin and found that changes in the number of protein peaks during the treatment course was significantly greater in patients who responded to the treatment compared to non-responders [72]. The therapeutic effect could be predicted with an AUROC of 0.75 using a differentiation method based on a combination of the peak levels of 2 proteins, fibrosis stage, and viral genotype. Therefore, proteomic analysis may also allow prediction of therapeutic effects and identification of proteins related to these effects, in addition to diagnosis of liver diseases.

## Conclusion

In recent decades, proteomic technologies based on mass spectrometry have been developed, and the reliability of these technologies continues to improve. Such advancements in proteomic techniques could contribute to the discovery of clinically useful biomarkers and the elucidation of the molecular mechanisms involved in disease pathogenesis. However, such advanced techniques are not necessarily utilized broadly and effectively because of the costs associated with the introduction of these technologies and the conscious differences that exist between developers and users of the application of identified biomarkers in clinical practice. Thus, as developers it is essential to make it clear as to how to use identified biomarker candidates appropriately.

In this review, we provided a survey of recent advances of proteomic investigations and several findings focused in liver diseases, including NAFLD, viral hepatitis, hepatic fibrosis, liver cirrhosis, and HCC. A low correlation between mRNA and protein expression levels has been found using exhaustive protein expression analysis [73]. Compared to detection of gene expression using DNA microarray analysis, techniques such as time-of-flight MS used in proteomics have relatively weak reproducibility and operability, and have not been developed sufficiently to allow wide use at all facilities. However, analysis of changes in protein expression is essential to investigate pathological conditions and reactions in vivo because processes at the organ, tissue and cellular levels are mostly regulated by proteins. About 20 proteins, including albumin and immunoglobulin, account for 99% of total serum protein, and proteins that may serve as biomarkers are present in trace amounts that account for the remaining 1%. Therefore, a more sensitive detection system to search for biomarkers is required, and this may allow discovery of clinically useful markers for all liver diseases. In addition, clarifying the profile of glycol- and phosphor-proteins may also be very important in understanding the pathogenesis of HCC and other liver diseases. The detection of such post-translational modification of proteins may reflect the pathogenesis of disease states more sensitively and specifically than methods that only examine the fluctuation of protein expression, as the profile of glycol- and phosphor-proteins in cancer cell-surface and -secreted protein are distinct from those in normal cells.

Proteins are assumed to be key molecules that define the characteristics and dynamics of cells and control biological reactions. Therefore, investigation of changes in protein expression levels is very important in understanding disease pathology. Further advances in proteomics techniques and establishment of simple and quantitative performance comparable to that of DNA microarrays are likely to promote proteomic studies and lead to further breakthroughs in clinical proteomics.

## Competing interests

The authors declare that they have no competing interests.

## Authors' contributions

HU carried out the interpretation of the data and preparation of the manuscript. SK and YT had contributed to the manuscript preparation. HT has contributed to the overall conception and critical review of the manuscript. All authors read and approved the final manuscript.
